# Supplementary material for: Identification of Key Metabolic Pathways and Biomarkers Underlying Flowering Time of Guar (Cyamopsis tetragonoloba (L.) Taub.) via Integrated Transcriptome-Metabolome Analysis
Source: Genes (Basel). 2021 Jun 22;12(7):952. doi: 10.3390/genes12070952 (PMC8303896; doi:10.3390/genes12070952)
Supplement: Supplementary file 1 [file genes-12-00952-s001.zip › genes-1196441-supplementary-figures-final.pdf]

genes

MDPI

Article

Identification of Key Metabolic Pathways and Biomarkers Underlying Flowering Time of Guar (*Cyamopsis tetragonoloba* (L.) Taub.) via Integrated Transcriptome-Metabolome Analysis

Elizaveta Grigoreva [1,2,3], Alexander Tkachenko [1*], Serafima Arkhimandritova [4], Aleksandar Beatovic [1], Pavel Ulianich [5], Vladimir Volkov [2,3], Dmitry Karzhaev [3], Cécile Ben [6], Laurent Gentzbittel [6] and Elena Potokina [2,3]

Supplementary Figures

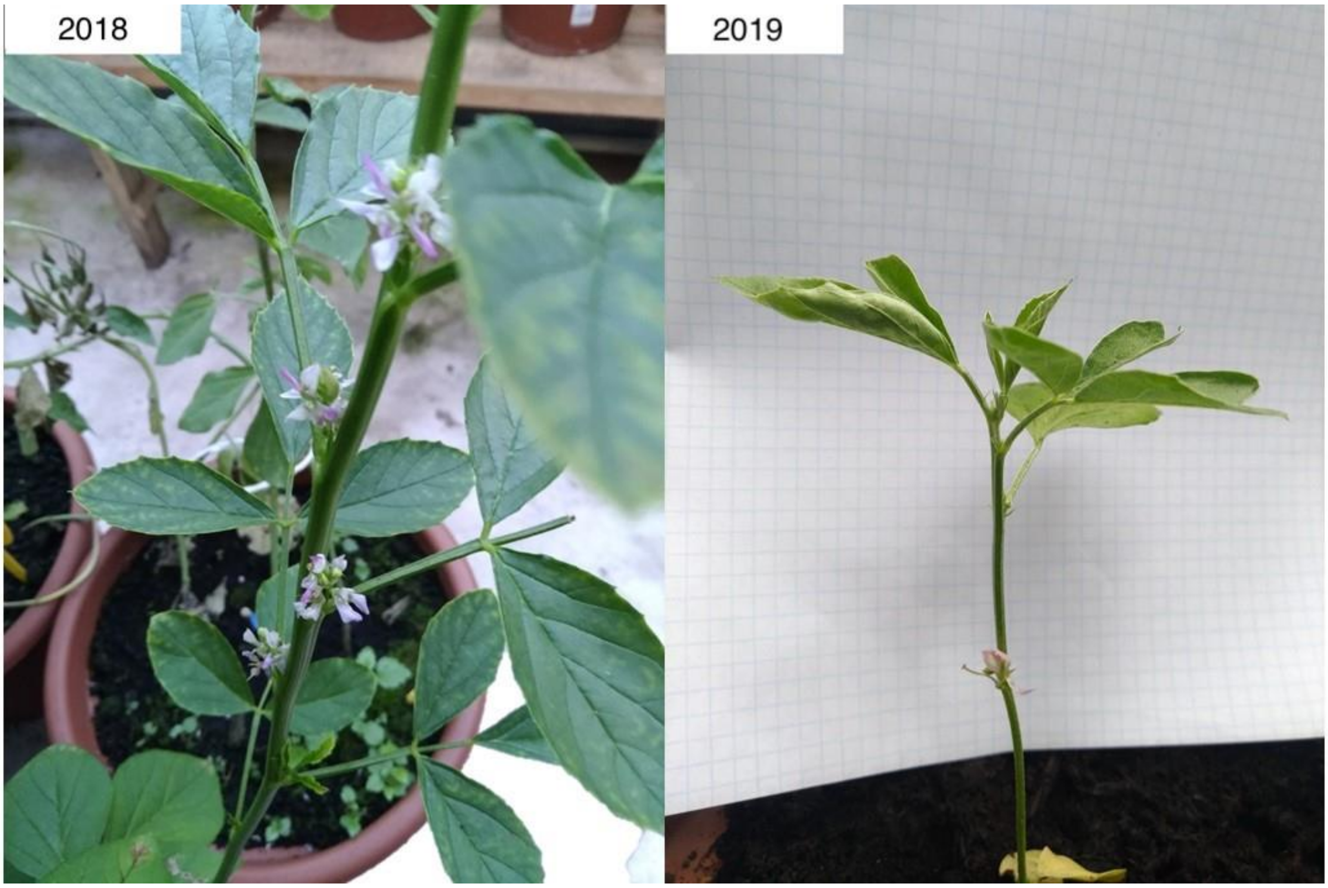

Figure S1. Photos of the plants of the same development stage in the conditions 2018 study and 2019 study.

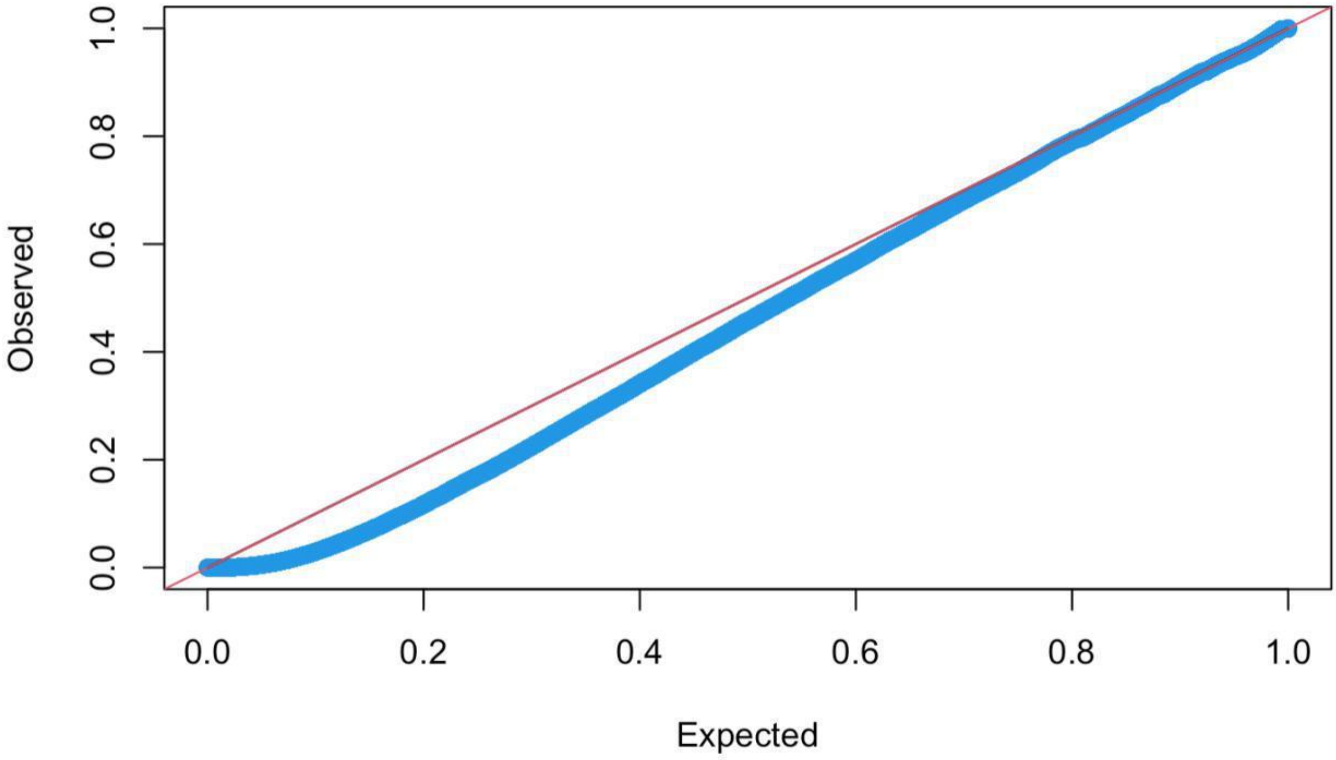

Figure S2. Q-Q plot of visual checking of *p*-values distribution.

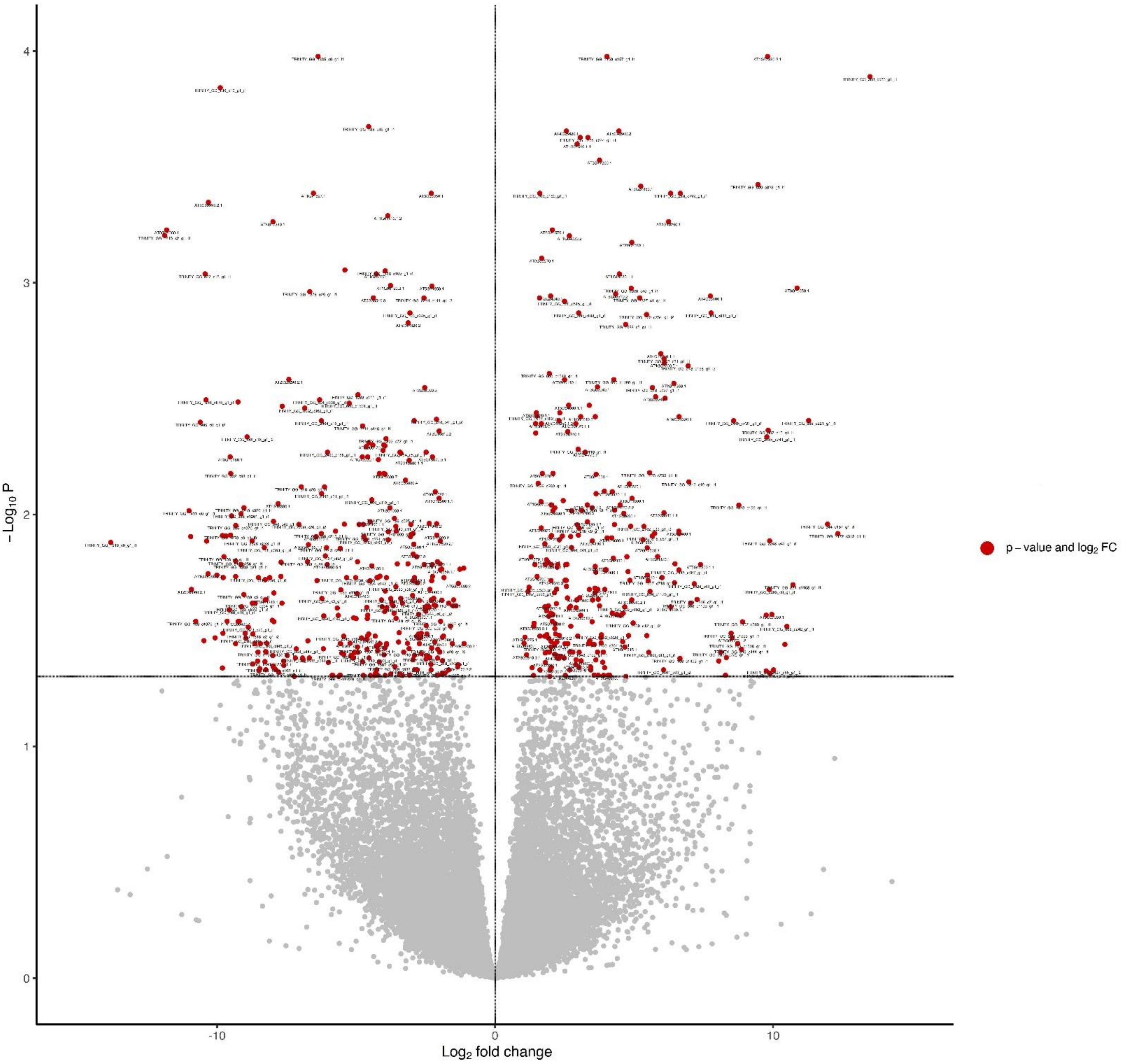

Figure S3. Volcano plot with highlighted significant DE transcripts selected by p.value and log2(FC) criteria.
