# Supplementary material for: Identification of Key Metabolic Pathways and Biomarkers Underlying Flowering Time of Guar (Cyamopsis tetragonoloba (L.) Taub.) via Integrated Transcriptome-Metabolome Analysis
Source: Genes (Basel). 2021 Jun 22;12(7):952. doi: 10.3390/genes12070952 (PMC8303896; doi:10.3390/genes12070952)
Supplement: Supplementary file 1 [file genes-12-00952-s001.zip › Supplementary description.pdf]

Supplementary name	Supplementary description
Supplementary Table1.csv	The table contains 63 metabolites from this study (2019) with crossed metabolites from a previous study (2018) with KEGG identifiers and statistical parameters.
Supplementary Table2.csv	Information about the total number of reads for each sample before and after the filtration procedure and GC content.
Supplementary Table3.csv	Information about BUSCO runs for each constructed assembly (Trinity genome guided, Trinity *de novo* 25mer, Trinity *de novo* 32mer, rnaSPAdes).
Supplementary Table4.csv	The counts depth for each of the 15 samples under the study.
Supplementary Table5.csv	The table contains list of top upregulated (overexpressed) transcripts (533) selected by p.adjusted values < 0.05.
Supplementary Table6.csv	The table contains list of top downregulated (overexpressed) transcripts (534 selected by p.adjusted values < 0.05.
Supplementary Table7.csv	The table contains all transcripts with blast hits against *Arabidopsis thaliana.*
Supplementary Figure1.jpg	Photos of the plants of the same development stage in the conditions 2018 study and 2019 study.
Supplementary Figure2.jpg	Q-Q plot of visual checking of p.values distribution.
Supplementary Figure3.jpg	Volcano plot with highlighted significant DE transcripts selected by p.value and log2(FC) criteria.
