# Supplementary figures and images for: Identification of Key Metabolic Pathways and Biomarkers Underlying Flowering Time of Guar (Cyamopsis tetragonoloba (L.) Taub.) via Integrated Transcriptome-Metabolome Analysis
Source: Genes (Basel). 2021 Jun 22;12(7):952. doi: 10.3390/genes12070952 (PMC8303896; doi:10.3390/genes12070952)

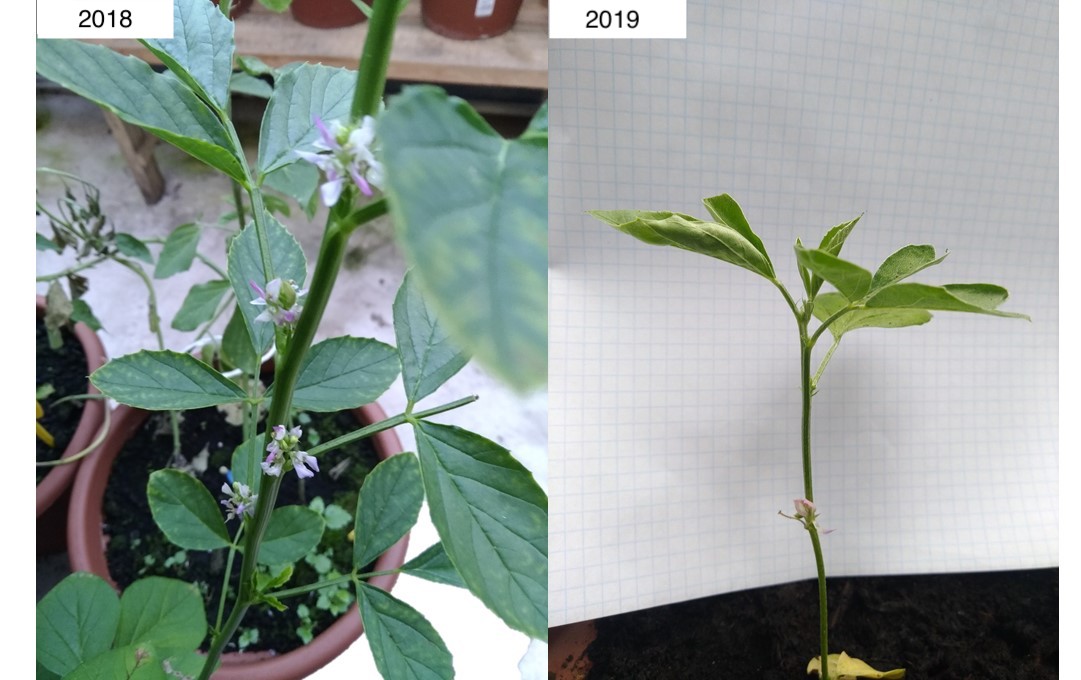

Supplement: Supplementary file 1 [file genes-12-00952-s001.zip › Supplementary Figure1.jpg]

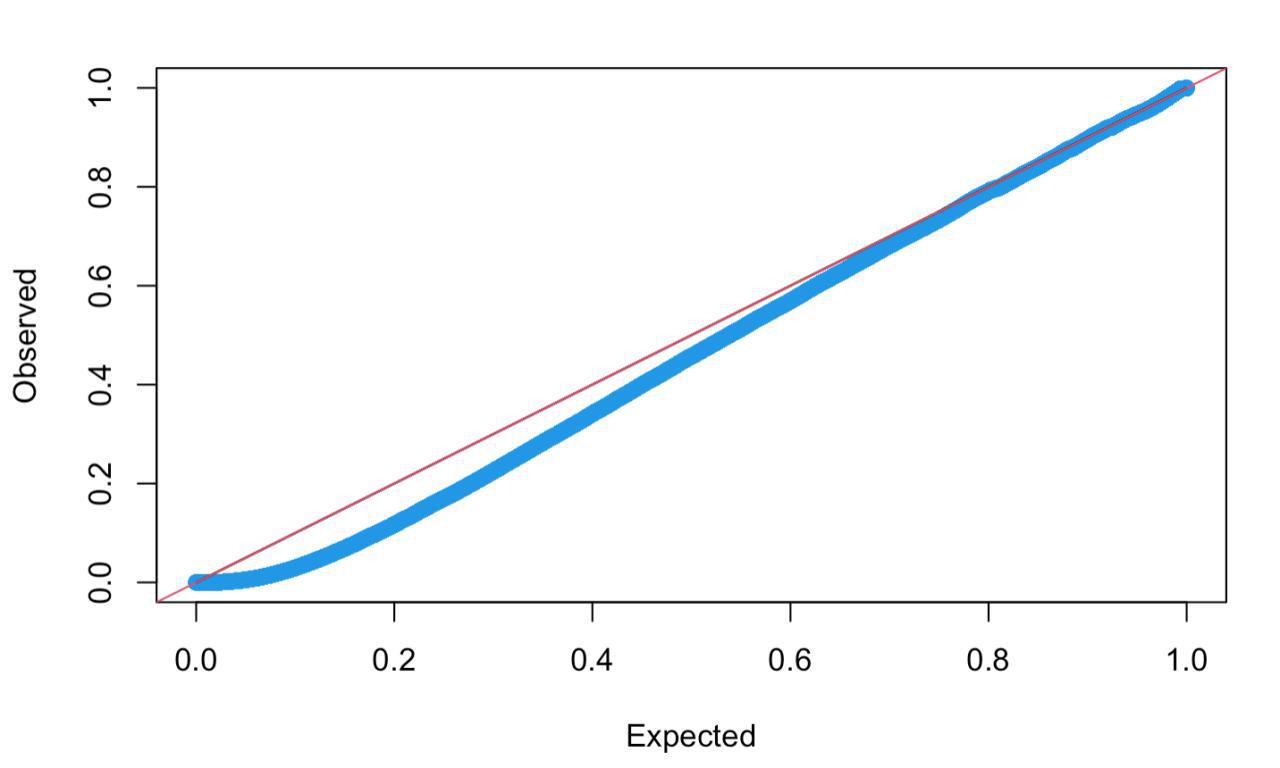

Supplement: Supplementary file 1 [file genes-12-00952-s001.zip › Supplementary Figure2.jpg]
